# Shadow Variation in Porcelain Inlay

**Published:** 1904-10

**Authors:** J. L. Newborn

**Affiliations:** Memphis, Tenn.


### SHADOW VARIATION IN PORCELAIN INLAY.

By J. L. Newborn, D. D. S., Memphis, Tenn.

(Written for *The American Journal of Dental Science.*)

For fifty years the dental profession has labored and experimented with cements and enamels, stimulated by one great desire and belief that finally it would be rewarded by the production of a filling material that would perfectly replace lost portions of human teeth.

With all the artistic skill in blending the white, pearl, blue, gray and yellow shades of both chloride and oxide of zinc cements, and all methods of hardening those materials, the result was a keen disappointment, because it was, after all, a flat opaque patch which lasted only a few years.

As the process of discovery progressed in the direction of the aesthetic demands for a more perfect compliance with nature in these respects, evolution naturally led us to the conclusion that translucent lifelike expression could only be produced in fused porcelain. Hence the result—porcelain moulded to shape, shaded to match color and cemented in position to restore lost parts of a tooth. The delicate and dainty process of baking these little bits of porcelain could not have been done in the cumbersome old coke-heated furnaces of the past.

Now the simplicity and compactness of modern furnaces so cheapens and abbreviates the whole process of baking an inlay that labor seems turned into play, or at least, dignified by the appearance of elegant ease.

As simple as it may seem, few dentists become experts in this class of work. Possibly the majority may succeed fairly well in filling teeth with gold and amalgam, constructing a bridge or making a set of teeth. These operations are mainly mechanical, easy to master by anyone who is fairly endowed with a talent for mechanism, but it takes an artist to blend the colors in inlay so perfectly to match the shading of the natural tooth that the joint between them can scarcely be discovered and to bake it so as not to burn out these colors.

If you can paint a landscape true to perspective, harmonious in the blending of lights and shadows, or copy the blush and almost the fragrance of the rose, then you may hope to become eminently successful in inlay work. If not, don't bring the highest style of dental art into disrepute by making a botch of an operation which you have neither the talent nor skill to perform.

In the hands of the artistic, the gifted, the porcelain inlay will no longer be looked upon with suspicion as a fad, but that it is a realization of ideals and principles that are born to live. Even in the hands of these scientific, artistic, experts, these ideals and living principles are not always realized, but finished results in a great majority of cases are disappointing and unsatisfactory. This apparently insurmountable obstacle to perfect results is known as "Shadow Variation."

Dr. Head, than whom no greater authority has written on porcelain inlay, says "that Shadow Variation will be a problem to the end of time, because no matter how well the porcelain inlay is matched, the shadow that comes from cementing pieces of porcelain into approximal cavities will always produce changes that are difficult to foresee." * * * * "And I am free to say now," he continues, "that until we get an absolutely perfect cement that will have the same light refraction as the tooth structure, the shadow variation will always be an obstacle that will interfere with the artistic appearance of the inlay. For instance, if one sits under the electric light so that the light shines from above on a tooth tipped with porcelain, the light is shut off by the cement from the porcelain beneath, causing a decided line of shadow, in a similar manner if the light is thrown from below on the porcelain tip there will be the same shadow variation between the tooth and the inlay. But since ordinarily the line of cement is perpendicular and the light comes direct to the person, if the inlay is well matched there will be no shadow variation."

Dr. Head blames the *cement* for the whole trouble, and says that "it will exist to the end of time unless we can get an absolutely perfect cement with the same light refraction as the tooth," and yet admits that two equally well matched inlays cemented with the same cement in the labial and approximal surfaces of a central incisor—the approximal cavity viewed obliquely will always have the shadow variation, but the one in the labial surface viewed vertically will have none.

It is a fact that phosphate of zinc cement, no matter how thin the stratum, is opake and absorbs all the light rays and transmits none, therefore not translucent, if not translucent it has no power of refrangibility.

I have been experimenting for four or five years with the Acetone solution of celluloid. Recently I ordered from the celluloid factory in New York samples of transparent, white, pearl, gray, and pink celluloid. With a creamy solution of these samples, I cemented inlays in different positions in natural and artificial teeth and proved conclusively that the cement which was perfectly transparent, and the gray or white or pink which were semi-translucent, all had the same lack of light refraction as that which Dr. Head attributes to the phosphate of zinc cement. If, then, the opake cement, the transparent cement, and the semi-translucent cements are all equally ineffectual in our hands to modify or obviate shadow variations in our inlays, I would like to ask Dr. Head where he expects to find "an absolutely perfect cement that will have the same light refraction as the tooth structure."

We see objects by the light which is reflected from them to the eye. Webster says "Refraction is the change in the direction of a ray of light where it enters obliquely a medium of different density from that through which it has previously moved."

In studying a question like this we must remember and be governed by the following laws of physics:

"When a ray of light passes perpendicularly from one medium to another it is not refracted.

"When a ray of light passes obliquely from a less refractive to a more refractive medium, it is refracted or bent toward the perpendicular.

"When a ray of light passes obliquely from a more refractive to a less refractive medium, it is refracted or bent from the perpendicular."

We must also remember that the crystalline structure of tooth enamel is a translucent and denser medium than the air through which rays of light pass to the eye, and that the vitrified structure of a fused porcelain inlay cemented in a cavity in a tooth is also a translucent and denser medium than the air through which rays of light pass to the eye.

If the inlay is cemented in the labial surface of a central incisor and viewed perpendicularly from the front, there would be no refraction, consequently no shadow variation—and it would be almost impossible to discover the inlay if it and the tooth were perfectly matched in color. But if the inlay is cemented in an approximal cavity or in any other position where it would be viewed obliquely, we *invariably* have that shadow which ruins the operation—no matter how perfectly the inlay and surrounding tooth were matched in color.

I do not believe that it is the refracted light from the inlay that produces the shadow, nor is it produced bv the refracted light from the tooth enamel surrounding the inlay.

The vitrified structure of the inlay and the crystalline structure of the enamel around it are different in density, so that rays of light reflected obliquely through both of these different media, through the air as a common medium, are reflected unevenly, the vibrations of the light waves are crossed, the image blurred and the shadow is the result.

Now if I have made it plain to you that the cement has nothing to do with the shadow business, but that it is the difference in the refrangibility of the inlay and the tooth structure surrounding it, how can the trouble be overcome or obviated unless you can change the vitrified structure of tooth enamel which surrounds it?

You will agree with me that this cannot be done.

Then, in the last analysis of this perplexing question, it seems to me we have it simmered down to one last and only alternative, and that is—by the use of mineral paints, to shade the inlay so that after it is burned in it will perfectly match the tooth.

Then we will have an inlay whose light rays are reflected from its surface through one medium that cannot blur or shade the image thrown from the tooth whether they are viewed obliquely or vertically.

---

### THE CHILD IN DENTISTRY.

By Bessie Burns Bennett, D. D. S.,
Baltimore, Md.

(Written for *The American Journal of Dental Science.*)

